# Toxicité au méthotrexate au cours d'une leucémie aiguë lymphoblastique

**DOI:** 10.11604/pamj.2014.17.16.2681

**Published:** 2014-01-15

**Authors:** Mouna Lamchahab, Said Benchekroun

**Affiliations:** 1 Service d'Hématologie et d'Oncologie Pédiatrique, Hôpital 20 Août 1953, CHU Ibn Rochd, Casablanca, Maroc

**Keywords:** Toxicité, méthotrexate, leucémie aiguë lymphoblastique, Toxicity, methotrexate, acute lymphoblastic leukemia

## Image in medicine

Mlle K.M, âgée de 19 ans, est suivie au service d'hématologie pour une LAL diagnostiqué sur les données cliniques (syndrome d'insuffisance médullaire, un syndrome tumoral important) et paracliniques (hyperleucocytose majeure à 145 720/mm3 et 95% de blastes). Le myélogramme et l'immunophénotypage confirmait le diagnostic de LAL biphénotypique T et B. Le caryotype était complexe. La patiente a été traitée selon le protocole national des LAL (protocole MARALL) avec une rémission complète après l'induction. Suite à son traitement de consolidation par MTX à haute dose (5g), la patiente présentait une fièvre à 40°c, une mucite grade 4 avec des ulcérations buccales douloureuses à surface fibrineuse, touchant le palais et la muqueuse jugale et débordant sur les lèvres empêchant l'alimentation. Ont noté également, une mélanodermie diffuse, hépato-splénomégalie et colite toxique (diarrhées profuses). Le bilan biologique objectivait une aplasie profonde, insuffisance rénale et cytolyse hépatique. Le diagnostic de toxicité grave au MTX cutanée, hématologique et viscérale a été retenu. Le traitement a consisté en une hydratation, alimentation parentérale, repos digestive, antalgiques, antibiotiques et acide folinique avec une très bonne évolution. Actuellement la patiente est en rémission complète maintenue. Le MTX est un cytostatique de la famille des antifolates, utilisé à haute dose en onco-hématologie. Ses effets secondaires sont dose-dépendants. Différents organes peuvent être touchés. La thrombopénie et la leucopénie sont les signes les plus précoces suivies de la stomatite ulcéreuse. Les signes cutanés sont à type de réaction anaphylactique, érythème et desquamation palmo-plantaires, associés à une mucite importante, un décollement bulleux voire une érythrodermie.

**Figure 1:**
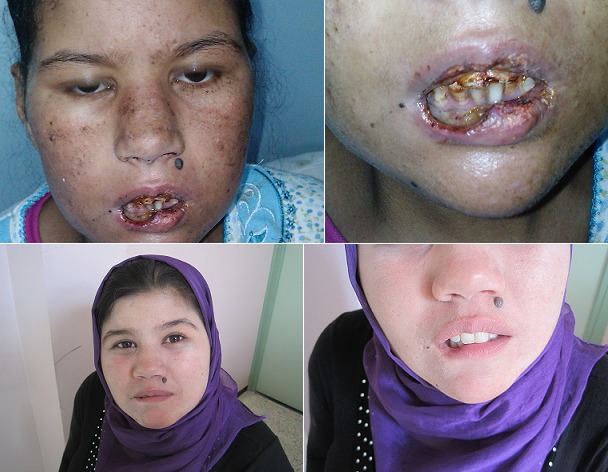
En haut: ulcérations buccales et chéilite érosive; En bas: amélioration après traitement

